# Nanoparticle Encapsulation for Antiretroviral Pre-Exposure Prophylaxis

**DOI:** 10.15436/2377-1372.17.1583

**Published:** 2017-08-07

**Authors:** Karl Khandalavala, Subhra Mandal, Rachel Pham, Christopher J. Destache, Annemarie Shibata

**Affiliations:** 1 Department of Biology, Creighton University, Omaha, NE, 68178, USA; 2 School of Pharmacy and Health Professions, Creighton University, Omaha, NE, 68178, USA

## Abstract

HIV continues to be one of the greatest challenges facing the global health community. More than 36 million people currently live with HIV and, in 2015 2.1 million new infections were reported globally. Pre-Exposure Prophylaxis (PrEP) prevents HIV infection by inhibiting viral entry, replication, or integration at the primary site of pathogenic contraction. Failures of large antiretroviral drug (ARV) PrEP clinical trials indicate the current insufficiencies of PrEP for women in high-risk areas, such as sub-Saharan Africa. A combination of social, adherence, and drug barriers create these insufficiencies and limit the efficacy of ARV. Nanotechnology offers the promise of extended drug release and enhances bioavailability of ARVs when encapsulated in polymeric nano-particles. Nanoparticle encapsulation has been evaluated *in vitro* in comparative studies to drug solutions and exhibit higher efficacy and lower cytotoxicity profiles. Delivery systems for nanoparticle PrEP facilitate administration of nano-encapsulated ARVs to high-risk tissues. In this mini-review, we summarize the comparative nanoparticle and drug solution studies and the potential of two delivery methods: thermosensitive gels and polymeric nanoparticle films for direct prophylactic applications.

## Introduction

Over the last fifteen years, international initiatives have designed more potent, new antiretroviral drugs(ARV) to reduce HIV infection and HIV-related deaths^[1]^. As a result, between 2000 – 2015, HIV infection rates fell 35%, and Acquired Immune Deficiency Syndrome (AIDS)-related deaths fell 27%^[1]^. Despite these recent successes, HIV infection and AIDS continue to be a challenging healthcare problem in the 21st century. According to a report by the American Foundation for AIDS Research, over 36 million people worldwide continue to live with HIV-1 and 2.1 million new HIV infections were reported in 2015^[1,2]^. Of those newly infected individuals, 47% were women and 8% were children less than 15 years old^[1]^. Young people between the ages of 15 and 24 accounted for 35% of all new adult infections, with infection rates of young women in this age group accounting for 20% of the global sum of HIV infections^[1,2]^ In sub-Saharan Africa, 15 – 24 year old females are eight times more likely to be infected with HIV than their male counterparts^[2]^. Greater than 80% of HIV infections are contracted through sexual transmission and 86% of female transmission has been attributed to heterosexual intercourse^[3,4]^. Factors such as mode of viral transmission, female physiology, and social, economic, and legal disadvantages contribute to increased rates of HIV infection in women. HIV/AIDS remains the leading cause of death for pre-menopausal women worldwide^[5]^. Given international efforts to reduce the annual global HIV infection rates by 90% by 2030^[1]^, highly efficacious therapeutic and preventative HIV therapeutic options must be available to at-risk populations, particularly women.

Post-infection HIV treatment using daily, highly active oral delivery of combination antiretroviral drug (ARV) therapies has significantly reduced HIV infection rates when such oral therapies are readily accessible and are reviewed elsewhere^[6]^. ART has been shown to significantly decrease the incidence of HIV transmission among serodiscordant couples when therapies are followed consistently by patient populations^[7]^. However, current oral therapies for (PrEP) are costly and are often in limited supply in developing countries and/or to at-risk populations^[8]^. Pre-Exposure Prophylaxis (PrEP) holds the promise of eliminating new infections and thereby the associated risks of HIV infection. Consequently, there is a need for the development of innovative, cost-effective, and highly efficacious PrEP. Nanotechnology has garnered considerable interest in the field of HIV PrEP because of its potential to extend the release, target and increase cellular uptake, and improve the chemical, enzymatic and metabolic stability of therapeutic drugs^[9,10]^. Various types of nanocarriers such as dendrimers, liposomes, polymeric nanoparticles and nanosuspensions are being evaluated for PrEP^[11]^. Vaccines are another promising area of innovative PrEP research and development but are beyond the focus of this review. This mini-review article presents the history of and the latest development in those nanofabrications showing promise for female PrEP with specific emphasis on nanoparticle fabrications involving antiretroviral drugs (ARVs).

### Use of antiretroviral therapy (ART) for PrEP in females

For a decade, it has been recognized that new infections of HIV must be reduced and that effective PrEP will be required to reach the worldwide goals for reducing the number of HIV infected individuals. The objective is to design PrEP that will block HIV at the mucosal membrane without causing tissue irritation or carrying the risk of developing resistance to ARVs. In order to eliminate the possibility of resistance, initial PrEP design involved the use of vaginal microbicides such as detergents, polyanionic inhibitors, or pH buffers that did not contain ARVs. Macromolecular entry inhibitors were largely unable to block HIV infection^[12–14]^. As concerns over drug-resistance waned^[15]^, and awareness that women need access to cost-effective HIV prevention strategies increased, investigators focused on ARV-mediated prevention. ARVs acting before integration of viral genomic material into the host cells became the strongest candidates for preventative treatments^[4,11]^. Initially, two classes of reverse transcriptase inhibitors, nucleoside reverse transcriptase inhibitors (NRTIs) and non-nucleoside reverse transcriptase inhibitors (NNRTIs), were the focus of PrEP efforts/strategies. Two nucleoside reverse transcriptase inhibitors, tenofovir disoproxil fumarate (TDF) and emtricitabine (FTC) were delivered orally for PrEP. Daily oral regimen of TDF demonstrated a 48.9% reduction in HIV infection among injection-drug users^[19]^. Daily oral regimen of TDF/FTC (Truvada) showed similar reduction in the incidence of HIV infection (44%) in men who have sex with other men^[20,21]^. The high efficacy of Truvada (44 – 75%) seen in clinical trials led to Truvada’s approval for PrEP in both men and women by the US Food and Drug Administration in 2012^[18,20,22–24]^. Importantly, these clinical trials established a correlation between plasma drug levels and prophylactic capacity of ARV PrEP. Seroconversions are frequently associated with low plasma drug concentrations in treatment groups suggesting that maintenance of plasma drug levels is important for PrEP efficacy^[25]^. Low plasma drug levels associated with a lack of adherence and observed in the FEM-PrEP and VOICE clinical trials where adherence was below 40% resulted in reduced efficacy of TDF PrEP^[26]^.

TDF was also formulated into a vaginal gel and evaluated for pharmacokinetic, safety, and antiviral efficacy^[16–18]^. The focus of the well-known VOICE trial was to assess the effectiveness of daily treatment with vaginal TDF gel and oral TDF and oral TDF/FTC (Truvada) in preventing sexually transmitted HIV-1 infection in women. Results from VOICE reinforce the importance of adherence to PrEP regimens. No significant difference was seen between drug and placebo treatments either vaginally or orally. Drug levels detected in the blood were low or absent for the majority of participants unless they were older than 25, married, or their sexual partner was older than 28^[23]^. CAPRISA 004 clinical trial investigated the efficacy of 1% TDF gel for PrEP revealing similar adherence-mediated effects. In this case, drug delivery specified for pre- and postcoital gel applications found the gel to be 39% effective in preventing HIV infection and concluded an overall efficacy of 54% in cohort with greater than 80% adherence. A subsequent study carried out over 2.5 years in 9 locations across South Africa demonstrated that adherence of high-risk female populations (> 70%) exhibited enhanced PrEP for HIV, but only 20% of the overall sample size was in this cohort^[27]^. More recently, three double-blind placebo controlled randomized trials demonstrate that daily oral TDF-based PrEP is quite successful given adherence and detectable TDF-blood levels^[28]^. These three studies found that daily oral PrEP reduced the risk of HIV infection in women. The Partners PrEP study included 1785 Kenyan and Ugandan women with HIV-infected partners. PrEP efficacy was 66% and 71%^[29]^. In the TDF2 study conducted in Botswana among heterosexual men and women, efficacy was 49% with a small sample size of 557 women^[18]^. A tenofovir study in Bangkok (BTS) showed that PrEP reduced the risk of HIV infection in women by 79%^[19]^. All five studies demonstrate that acquisition of HIV occurs during periods of low or no adherence to PrEP.

Taken together, these studies demonstrate that participant adherence directly influences PrEP efficacy. Surmounting the adherence barrier necessitates the development of cost-effective, easily used, and long-lasting PrEP fabrications.

### ARV loaded Polymeric Nanoparticles

Polymeric nanoparticles for ARV drug delivery can encapsulate various drug formulations for selective and enhanced drug delivery. Polymeric NP pharmacokinetic and material development are reviewed elsewhere^[12,30]^. Nanoparticle sustained drug delivery is likely to reduce the required frequency of drug application for proper efficacy. Reducing dosing complexities and frequency are likely to increase treatment adherence and effectiveness while decreasing cost and high dosage toxicity. Current developments of ARV-encapsulated NP treatments for PrEP typically utilized poly(lactic-co-glycolic acid) (PLGA) based prophylactic modalities. Other polymers such as Cellulose Acetate Phalate (CAP) and Polycaprolactone (PCL) are being explored. Specific polymers, such as CAP, have anti-microbicidal function and may serve not only as a nanoparticle polymer but also enhance PrEP efficacy.

PLGA is a common nanoparticle polymer. Studies suggest that PLGA-NPs undergo endosomal uptake allowing for delivery of encapsulated drug directly to cellular cytoplasm and thus enhancing ARV drug uptake into the cell^[2,3]^. Cellular *in vitro* assays examined the efficacy of ARV in solution verses encapsulation of ARV in PLGA-NPs. In many studies, encapsulation of ARV in PLGA-NP has shown increased prophylactic efficacy of PLGA-ARV-NP as compared to ARV in solution alone. Mandal *et al*., 2016 encapsulated FTC in PLGA-NPs via a water-in-oil-in-water emulsion method^[31]^ (Table 1). *In vitro* TZM-bl and human Peripheral Blood Mononuclear Cell (PBMC) assays demonstrated that PLGA-FTC-NPs significantly reduced FTC IC_50_ levels against HIV as compared with FTC solution^[31,32]^. PLGA-FTC-NPs protected PBMCs for up to 21 days post-HIV exposure^[32]^.

Chaowanachan *et al*., 2013 examined the encapsulation of low-solubility ARV drugs. Efavirenz (EFV), a non-nucleoside reverse transcriptase inhibitor (NNRTI), and saquinavir (SQV), a protease inhibitor, were encapsulated in PLGA-NP. PLGA-EFV-NP were loaded using single solvent emulsion evaporation fabrication^[33,34]^. PLGA-SQV-NP were formulated by nanoprecipitation^[35]^. Individually, PLGA-EFV-NP and PLGA-SQV-NP showed increased efficacy over their respective free-drug solutions in TZM-bl indicator cell assays following 24hr pretreatment. PLGA-EFV-NP and PLGA-SQV-NP showed significantly reduced ARV IC_50_ levels against HIV as compared to drug in solution (Table 1). Synergistic effects with free tenofovir (TFV) were also evaluated using TZM-bl cells by applying 1:1 equimolar IC_50_ drug concentrations at free EFV/SQV:TFV and PLGA-EVF/PLGA-SQV:TFV ratios. PLGA-EFV/TFV showed a 3-fold decrease in combined IC_50_ and PLGA-SQV/TFV exhibited a 20-fold decrease in combined IC_50_ over their respective free drug treatments^[35]^ indicative of increased drug delivery to cells by nanoparticles.

To examine the potential synergistic anti-HIV activity of combination ART in nano-particle fabrications, encapsulation of single, dual or triple drug combinations of the entry inhibitor maraviroc, (MVC), the NNRTI etravirine (ETR), and the ISTI raltegravir (RAL) into PLGA-NPs were developed^[36]^ (Table 1). Drugs were encapsulated in PLGA using an emulsion-solvent evaporation protocol (Table 1). Single-encapsulation method avoids loading complexities often seen when nanofabricating drugs with different physiochemical profiles^[37]^. PrEP efficacy of PLGA-encapsulated treatments against HIV-BaL was examined using TZM-bl assays^[35]^. Only ETR-NP treatments exhibited decreased IC_50_ values while MVC-NP and RAL-NP had increased IC_50_ values compared to free-MVC/RAL. Tandem treatments of RAL-NP/MVC-NP, MVC-NP/ETR-NP, and ETR-NP/RAL-NP were evaluated using TZM-bl *in vitro* assays. Only ETR-containing fabrications showed improved efficacy, with 10-fold reduction in IC_50_ for MVC/ETR-NP (IC_50_:0.38nM) and RAL/ETR-NP (IC_50_: 0.40nM) paired treatments compared to the ETR solution (ETR-Sol) combined with MVC-Sol (IC_50_:3.02nM) and RAL-Sol (IC_50_: 4.21nM). ETR-NP combinations were also three times more efficacious in blocking cell-cell HIV transmission. Drug synergy was only observed when ETR was paired with RAL or MVC and encapsulated into a polymeric nano-formulation. Interestingly, triple NP treatments did not show any increased potency over the double drug combinations (IC_50_:0.40nM)^[36]^. However, triple combination NP treatments were associated with higher intracellular concentrations than free-drug triple combination as measured by LC-MS/MS. Triple combination NP treatments were also protective against RT-SHIV challenges in macaque cervico-vaginal explants tissue^[34]^. Differential encapsulation efficiencies for RAL were observed in fabrication of combination PLGA-EFV-RAL NPs. Oil-in-water emulsion with PLGA: Pluronic 127 at 1:2 w/w ratio resulted in EFV entrapment efficiency of 55.8% and RAL at 98.2% (Table 1)^[38]^. Despite different encapsulation percentages, the combination RAL-EFV-NPs showed lower inhibitory concentrations than drug solutions in *in vitro* TZM-bl assays (Table 1)^[38]^.

Multiple drug encapsulations of efavirenz (EFV) boosted by two protease inhibitors lopinavir/ritonavir (LPV/r) in PLGA-NPs using an emulsion-solvent-evaporation method with a high-pressure homogenization component to increase encapsulation efficiency were designed^[38]^. Encapsulation efficiency of EFV, LPV/r were 81.0, 79.8, and 79.5%, respectively (Table 1)^[38,42]^. TZM-bl cells inoculated with HIV-1_NL4-3_ after treatment with PLGA-EFV-LPV/r NPs 24 hr prior to infection. IC50 values for all encapsulated ARTs were in the nano-molar range (Ritonavir: 14.01nM, LPV: 16.54nM, EFV 30.73nM). EFV and LPV/r delivered by PLGA-NPs remained in different cellular compartments for as long as seven days in a HIV-1_NL4-3_ challenged human T cell line as determined by sub cellular fractionation and HPLC analysis. Only ritonavir solution was found at detectable levels in cells at seven days while combination NPs showed measurable drug levels in membrane, nuclear, and cytoskeletal fractions indicating sustained release of drug through NP delivery^[38,42]^.

Polymer alternatives to PLGA such as poly(ε-caprolactone) (PCL) surface coated NPs were shown to enhance encapsulated drug bioavailability and intracellular retention^[43–45]^. Addition of Poly-Ethylene Oxide (PEO), Sodium Lauryl Sulfate (SLS), and Cetyl-Trimethyl Ammonium Bromide (CTAB) were surface modifications compared in PCL-NP fabrications. Each fabrication encapsulated dapivirine (DAP), a NNRTI, using a modified solvent displacement method^[43,45]^ that yielded higher DAP association efficiencies (Table 1) than previously reported with PLGA^[46,47]^. In TZM-bl assays infected with HIV-1 BaL virus, PEO-PCL-DAP-NP was less efficacious than free dapivirine solution while the two other surface treatments (SLS/CTAB) demonstrated lower EC_50_ values. However, CTAB surface coatings were found to be 4-fold more cytotoxic than the free dapivirine solution. PEO/SLS-PCL demonstrated significantly less cytotoxicity than free drug. Anti-HIV efficacy of these NPs using PBMC assays challenged with HIV after 2 hrs NP treatment resulted in EC_50_ values in the nano-molar range, 3–7 fold lower than free drug^[47,48]^. PEO-PCL exhibited the lowest cytotoxic concentration (CC_50_) in PMBCs at 10^5^ nM, half that of SLS-PCL and approximately 200-fold less than CTAB. These NPs inhibited HIV infection of monocyte-derived dendritic cells (Mo-DC) and CD4+ T cell co-culture for 14 days^[49,50]^. Single drug applications to Mo-DC showed EC_50_ for NP-encapsulated DAP at 7–12 fold lower than free DAP. Cytotoxicity of NP treatments to Mo-DCs mirrored PBMC assays with CTAB-coated NP (PCL-CTAB) having the highest cytotoxicity (CC_50_: 1,728 ± 142nM), 20-fold less than PCL-SLS (CC_50_: 38, 442 ±7, 920nM), and 40-fold less than PCL-PEO (CC_50_: 76, 984 ± 8, 467nM)^[38]^. PEO-PCL-DAP NPs were chosen for *in vivo* pharmacokinetic analysis due to their enhanced inhibition of infection and comparably low cytotoxicity profile^[51]^. Application of PEO-PCL-DAP NP or free DAP solution intravaginally to female mice showed enhanced retention of NPs in vaginal fluid. PEO-PCL-DAP NP retained drug levels above the previously established drug level for DAP for 24hrs while free DAP solution maintained threshold levels for 4 hrs. These results indicate the extended protective capacity of DAP NPs *in vivo*^[51,52]^.

Another nanoparticle polymer under investigation for PrEP is Cellulose Acetate Phthalate (CAP). CAP is unique to other functionally inert polymers because CAP has anti-microbicial properties that inhibit HIV-1 entry. CAP has been shown to bind to gp 120 and to gp 41 on HIV and to form six-helix bundles with R4 and R5 tropic viruses^[53,54]^. CAP also may destroy viral particles by stripping envelope glycol-proteins and causing HIV^[53–55]^. CAP is pH a sensitive polymer that depolymerizes at pH higher than 6.2^[56]^. Since vaginal mucosal pH is lower than 6.2, CAP-NPs are likely to remain stable in the acidic pH environment. CAP-EFV-NPs were formulated by nano-precipitation method and yielded an EFV entrapment efficiency of 98.1% ± 1.2% (Table 1)^[57]^. Short term (4 hr) and long term (3 day) PrEP of CAP-EFV-NPs against HIV-1_NL4-3_ challenge were assessed *in vitro* using TZM-bl assays. CAP-EFV-NPs significantly reduced HIV infection at concentrations below 50 ng/mL compared to EFV drug solution^[57]^. At 3 days the EFV solution had significantly lower antiretroviral activity compared to CAP-EFV-NPs treatment at equivalent concentrations (5 ng/mL)^[57]^. CAP-EFV-NPs reduced the cytotoxicity of EFV on HeLa cells with significantly higher cell viability at 48 h and 96 h^[57]^. CAP may be another cost-effective polymer option for NP synthesis and PrEP.

### ARV loaded Polymeric NPs in TMS gel

**Figure 1:**
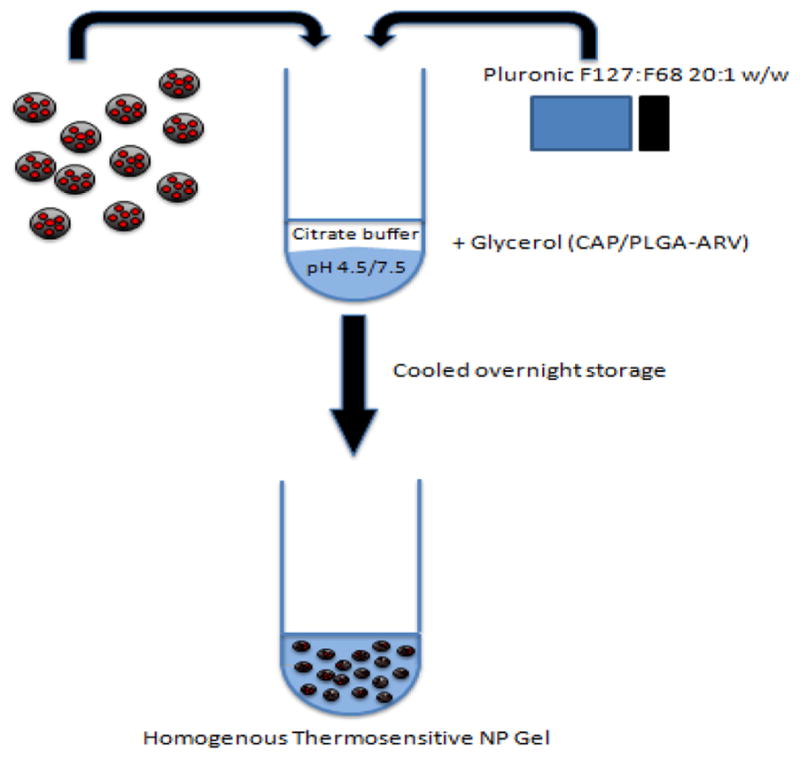
Fabrication of thermosensitive gel with NP-encapsulated ARVs for vaginal application of PrEP to high-risk tissues. ARVs encapsulated in polymeric nano-particles using oil-in-water emulsion technique with the organic phase comprised of ARVs, DMSO, N-methyl-pyrrolidone, and ethyl acetate emulsified in ultrapure water. ARV-NPs are prepared in a citrate buffer with the addition of Plurionic F127 and F68 at a 20:1 ratio to the buffer for gelation. The solution is set overnight in a cooled environment. pH modifications are made for CAP-NP and PLGA-ARV-NP fabrications along with glycerol addition. Black-NPs, Red: ARVs, Blue: polymer^[38]^.

For ease of topical application to reproductive tissues some NP fabrications have utilized thermosensitive (TMS) gels. Topical gels coupled with polymeric NP encapsulated ARVs may offer direct application to principal sites of HIV exposure prior to sexual intercourse, ensure uniform drug application, and control drug kinetics for elongated release. Thermosensitivity modulates rheological properties by increasing viscosity as a function of increasing body temperature upon application to facilitate delivery and enhance vaginal retention^[58,59]^. Mechanisms for gelation have been explored^[60]^. Osmolarity is an important consideration for gels as failures in large clinical trials including CAPRISA-004 have been attributed to hyperosmolar gels causing inflammation and increased susceptibility to HIV-1 infection^[61]^. Combinations of pluronic polymers (F127/F68) are used to tailor the rhelogical properties with citrate-buffered NP solutions, DMSO, and N-Methyl pyrrolidone^[38]^ (Figure 1). Recommended values of TMS gel fabrications are 380–1200 mOsm/kg^[62–64]^. Initial gel fabrications were analyzed using *in vitro* cell assays that determined the delivery/translocation of Rhodamine 6G labeled PLGA-NPs (PLGA-Rhod6G-NP) through thermosensitive gels into HeLa cells. Fluorescence of PLGA-Rhod6G-NP was observed in HeLa cells after 30 minutes of incubation showing rapid release and uptake of NPs into cells. Rhodamine 6G fluorescence was maintained for up to seven days *in vitro*^[38]^. PLGA-Rhod6G-NP delivered to the vaginal tissues of humanized mice showed uniform distribution in vaginal tissues. PLGA-Rhod6G-NP was specifically localized in the vaginal epithelium for up to 24 hours^[65]^. As proof-of-concept experiments, CAP-EFV-NP were incorporated into TMS and examined efficacy^[57]^. HIV-1_NL4-3_ antiviral efficacy was measured *in vitro* using TZM-bl assays following CAP-EFV-NP-TMS, CAP-NP-TMS, and EVF-TMS pre-treatment. TZMbl cells were challenged with HIV-1 four hours post-treatment and CAP-EVF-NP-TMS showed higher efficacy with 90% antiviral activity at 500pg/mL of EFV. These studies indicated enhanced efficacy of CAP-ARV-NP-TMS and expanded the study of TMS delivery to ARVs more likely to be used in human clinical studies^[57]^.

*In vivo* efficacy of PLGA-ARV-NP-TMS has been recently demonstrated in humanized mice. PLGA-Rilpivirine (RPV) –NPs were formulated by encapsulation using ion-solvent-evaporation technique for incorporation into TMS gel^[66]^. Kovarova *et al*., 2015 achieved 98% RPV association efficiency and embedded their NPs in 20:1 Pluronic F127:F68 ratio TMS gel^[65]^. Humanized BLT mice treated with PLGA-RPV-NP-TMS (17.5μg RPV) were completely protected when challenged with high dose HIV-1_RHPA_ 1.5 hrs post-application. Only half of these mice were protected from HIV challenge 24 h after application of PLGA-RPV–NPs-TMS (Table 1) as determined by vR-NART-PCR analysis. Further, vDNA analysis 7–8 weeks after the HIV challenge confirmed the seroconversion results from plasma vRNA by RT-PCR analysis^[65]^. Destache *et al*., 2016 examined TMS gel fabrication using PLGA-TDF –NPs. PLGA-TDF –NPs were encapsulated using an oil-in-water emulsification^[12]^. An additive ion-pairing agent yielded a TDF encapsulation efficiency of 52.9% (Table 1)^[67]^, significantly higher than the previously reported^[36,37]^ due to its water soluble properties. TMS was fabricated with the modification of neutral 7.4 pH PBS instead of citrate buffer^[50]^. TDF-NP-TMS gel with three concentrations of TDF (0.1%, 0.5%, 1% w/v TDF) were individually applied intra-vaginally to Hu-BLT mice. Following TDF-NP-TMS treatment mice were challenged with 2 transmission/founder HIV-1 strains at three time points. The four hour (n = 4) and 24 hour (n = 6) challenge groups showed 100% protection against HIV-1 challenge as determined by plasma viral load (pVL)^[67]^. All mice challenged at seven days showed HIV-infection at 14 days post-inoculation, signifying TDF-NP-TMS gel capacity for intermediate protective capacity, but, currently, not for longer time durations (>24hrs).

### ARV loaded Polymeric NPs in film

Films serve as another platform to enhance the topical delivery of ARVs encapsulated in NPs to primary sites of HIV-1 exposure. Films offer some advantages to gels since films avoid the need for an applicator and reduce leakage issues^[70]^. Clinical trials of film-encapsulated dapivirine indicated the efficacy of these treatments in maintaining plasma drug levels comparable to that of gel fabrications^[74]^. Using solvent casting with glycerin as a plasticizer, prepared films of PLGA/stearylamine (SA)-Tenofovir-NPs were investigated for efficacy^[71]^. PLGA/SA-Tenofovir-NPs were produced by double emulsion/solvent evaporation and demonstrated much higher NP-drug association efficiency (PLGA/SA: 53.5 ± 4.9%) than pure PLGA-based NPs (18.5 ± 2.5%)^[71]^. NP-embedded films were thicker and weaker than pure films, potentially complicating fabrication, handling, and applications, but they maintained minimum pharmaceutical thresholds. Like gels, the physiochemical properties of films must conform to physiologic osmolarity and pH levels to ensure safe vaginal applications^[70,71]^. PLGA/SA-Tenofovir-NP-film formulations were within physiologic thresholds^[71]^. Tenofovir release was sustained further in Tenofovir-NP-film fabrications compared to Tenofovir-NP and Tenofovir-film fabrications (Table 1).

Machado *et al*., 2016 examined PLGA-EFV-NPs embedded with tenofovir drug solution in polymeric films. PLGA-EFV-NPs were fabricated by emulsion-solvent-evaporation^[46]^ and encapsulation efficiency of EFV was high (Table 1)^[70,71]^. EFV exhibited sustained release *in vitro* from NP-film fabrication with 40% burst release at one hour and sustained release at 20% over the next 24h in simulated vaginal fluid (SVF). Non-gel EFV-NP treatments released at a much faster rate indicating the potential of the film-matrix to extend NP drug release in SVF. PLGA-EFV-NP/TFV-films examined *in vivo* using female CD-1 mice showed enhanced retention of TFV for two hours but overall rapid decreases in drug concentration. Similar decreases have been observed with intra-vaginal tablet-tenofovir drug formulations in macaques and rabbits^[75,76]^. EFV concentrations were also sustained at early time points (30 min) using NP-EFV/TFV-solution films compared to EFV solution/TFV solution-film formulations, indicating the ability of NPs in films to elongate drug release of both EFV and TFV^[71]^.

Film fabrication differing in PVA: HPMC polymer excipient ratio has been designed. Polymer film embedded with IQP-0528, an NNRTI with entry inhibiting capabilities, was encapsulated in PLGA-NPs (PLGA/Eudragit S100-IQP-0582-NPs) by double emulsion^[77]^. Films were optimized for physiologic physiochemical properties and drug loaded to 1.5% wt/wt (drug/film)^[73,78]^. *In vitro* drug release was measured in continuous flow in-line Franz cells^[78,79]^ and showed significantly elongated release of IQP-0528-NP from films (24hr: 51.65% ± 7.22% release) compared to free IQP-0528 films (1hr: 100% release). However, *in vivo* pharmacokinetic analysis on pigtailed macaques found that median drug levels at 24hrs were higher in the free-IQP-0528 films as opposed to the IQP-0528-NP-films (Table 1)^[72]^. All drug levels were well above IQP-0528 *in vitro* IC_90_ value (0.146 μg/mL)^[80]^ in the distal and proximal vaginal fluid indicating uniform coverage and enhanced retention of drug in the vaginal environment^[72]^.

Films have shown mixed results as a NP delivery modality. *In vitro* models with PLGA/SA-TFV NPs and PLGA-EFV-NPs both showed elongated release compared to free-drug film fabrications (2 film articles). However, *in vivo* pharmacokinetic studies using PLGA-IQP-0582-NPs exhibited drug clearance rates similar to that of the IQP-0582 molecule in solution. Currently, there are no studies directly comparing gels and films as delivery systems for ARV-NPs.

## Conclusion

Recent clinical studies have shown that PrEP can be highly efficacious given patient adherence. Widespread use of PrEP must be cost-effective and stable. New highly efficacious PrEP that can be delivered to at risk populations must be developed. Nanoparticle fabrications of ARVs delivered in thermosensitive gels or polymeric films may provide a means for low cost, highly effective PrEP and is an important goal of current PrEP research. There is an increased need for studies investigating new prophylaxis for women^[21]^. Prophylaxis that enhances current nanoparticle technology to deliver higher and sustained concentrations of ARV drugs is likely to provide enhanced efficacy. Future studies will show the viability of nanoparticle fabrications for PrEP.

## Figures and Tables

**Figure 2:**
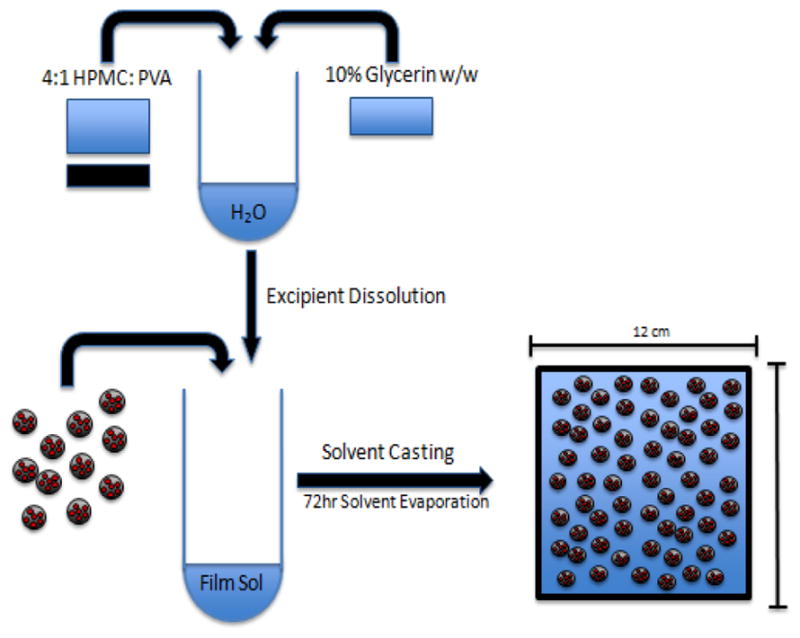
Fabrication of polymeric film with NP-encapsulated ARVs embedded into film for vaginal application of PrEP to high-risk tissues. Polymeric excipients HPMC and PVA were combined at a 4:1 ratio in a solution to 3% w/w of water containing 90% excipient polymers and 10% additional glycerin. ARV encapsulated PLGA NPs were added to the polymer-glycerin solution immediately before casting in 12 cm X 12 cm polystyrene molds^[70–73]^.

**Table 1:** Nanoparticle encapsulated ARV for PrEP *in vivo* and *in vitro* using film and gel delivery modalities.

Prophylactic Modality	ARV	Drug Target	Drug Target	EE% a/AE%^b^	Level of Study	Duration of measurement	Results
PLGA/SA	TFV	NRTI	Film	AE: 53.5% ± 4.9%^b^	SVF	24 h	PLGA/SA-TFV-Film sustained drug release (60% released in 24 hrs) (71)
PLGA	IQP-0528	NNRTI/EI	Film	NR	Macaques	24 h	*In vitro*: 51.65% ± 7.22% drug release at 24hrs; *In vivo*: (72)
PLGA	EFV-Free TFV	NNRTI-NRTI	Film	96.8 ± 2.5%	Female CD-1 Mice	24 h	*In vivo*: 2 hr. burst release, rapid drug decreases (70)
SLS-PCL	DAP	NNRTI	NP Solution	97.6% ± 0.4%	TZM/PBMC/Mo-DC--T4	14 d	TZM-bl: no EC_50_ difference; PBMC: 6.8-fold decrease EC_50_; Mo-DC: 12.6-fold EC_50_ decrease; moderate *in vitro* cytotoxicity (46)
CTAB-PCL	DAP	NNRTI	NP Solution	97.9% ± 0.3%	TZM/PBMC/Mo-DC--T4	14 d	TZM-bl: no EC_50_ difference; PBMC: 6.3-fold decrease EC_50_; Mo-DC:12-fold decrease EC_50_; high cytotoxicity in all *in vitro* assays (46)
PEO-PCL	DAP	NNRTI	NP Solution	97.6% ± 0.1%^b^	ICR mice-topical	24 h	TZM-bl: no EC_50_ difference; PBMC: 3.8-fold decrease EC_50_; Mo-DC: 6.81-fold EC_50_ decrease; *In vivo*: 9-fold increase DAP NP Vs. DAP Sol. At 24 hrs. (47)
PLGA	EFV-LPV/r	NNRTI-PI/PI	NP Solution	EFV: 79.5%, LPV: 79.8%, RTV:81%	TZM-bl indicator cells	48 h	Similar IC_50_ in combination NP than for each drug solution separately (42)
PLGA	EFV	NNRTI	NP Solution	44.5% ± 2.7%^a^	TZM-bl indicator cells	48 h	IC_50_: 54.6-fold decrease (35)
PLGA	SQV	PI	NP Solution	43.8% ± 15.2%^a^	TZM-bl indicator cells	48 h	IC_50_: 1.65-fold decrease (35)
PLGA	MVC-ETR-RAL	EI-NNRTI-IS-TI	NP Solution	91.0 ± 9.9, 16.8 ± 2.6, 12.0 ± 0.6^a^	TMZ-bl indicator cells	48 h	IC_50_: 8-fold decrease in combination treatment relative to the free drugs in combination with one another (36)
PLGA	FTC	NRTI	NP Solution	EE: 50.6 ± 5.5%^a^	TMZ-bl indicator cells	24 h	43-fold decrease in IC_50_ in the PLGA-FTC compared to the FTC solution (32)
PLGA	RPV	NNRTI	TMS	98 ± 0.7%^b^	Hu-BLT Mice-Topical	7–8 weeks	50% protection n = 12 (65)
PLGA	TDF	NNRTI	TMS	52.9%^a^	Hu-BLT mice-topical	4 weeks	100% protection 4,24, all infected at 7day time pt. (67)
CAP	EFV	NNRTI	TMS	EE: 98.1% ± 1.2%^b^	TMZ-bl indicator cells	3 d	Combination NP significantly higher % antiviral activity compared to EFV solution at same concentration. (57)

32, 35, 36, 42, 46, 47, 57, 65, 67, 70–72

a Encapsulation Efficiency = [(Drug_measured_)/(Drug_fabrication_)]*100

b Association Efficiency = [(Drug_Fabrication_ − Drug_measured_)/(Drug_Fabrication_)]

**Table 2:** Abbreviations.

Abbreviation	Name
ART	Anti-retroviral therapy
ARV	Anti-retroviral
AZT/ZDV	Zidovudine (NRTI)
EFV	Efavirenz (NNRTI)
3CT	Lamivudine (NRTI)
LMV	Lamivudine (NRTI)
Lf	Lactoferrin (NP)
PEO	Poly(ethylene oxide)
PCL	Poly(ε-caprolactone)
Hu-BLT	humanized bone marrow-liver-thymus mice
PLGA	Poly(lactic-co-glycolic acid)
TDF	Tenofovirdisoproxilfumarate (NRTI)
TFV	Tenofovir (NRTI)
LPN/r	Lopinavir/ritonavir PI
PI	Protease Inhibitror
IN	Integraseinihibitor
SQV	Saquinavir (PI)
MVC	Maraviroc (Entry inhibt)
ETR	Etravirine (NNRTI)
RAL	Raltegravir (IN)
TMS	Thermosensitive Gel
CAP	Cellulose Acetate Phthalate
FTC	Emtricitabine (NRTI)
RPV	Rilpivirine (NNRTI)
Dapivirine	Dapivirine (NNRTI)
EI	Entry Inhibitor
DAP	Dapivirine
